# Atg2: A novel phospholipid transfer protein that mediates *de novo* autophagosome biogenesis

**DOI:** 10.1002/pro.3623

**Published:** 2019-04-29

**Authors:** Takuo Osawa, Nobuo N. Noda

**Affiliations:** ^1^ Institute of Microbial Chemistry (BIKAKEN) Tokyo 141‐0021 Japan

**Keywords:** Atg2, lipid transfer protein, autophagy, autophagosome, isolation membrane, ER exit site

## Abstract

The degradation of cytoplasmic components via autophagy is crucial for intracellular homeostasis. In the process of autophagy, a newly synthesized isolation membrane (IM) is developed to sequester degradation targets and eventually the IM seals, forming an autophagosome. One of the most poorly understood autophagy‐related proteins is Atg2, which is known to localize to a contact site between the edge of the expanding IM and the exit site of the endoplasmic reticulum (ERES). Recent advances in structural and biochemical analyses have been applied to Atg2 and have revealed it to be a novel multifunctional protein that tethers membranes and transfers phospholipids between them. Considering that Atg2 is essential for the expansion of the IM that requires phospholipids as building blocks, it is suggested that Atg2 transfers phospholipids from the ERES to the IM during the process of autophagosome formation, suggesting that lipid transfer proteins can mediate *de novo* organelle biogenesis.

**Abbreviations**
Atg: autophagy‐related
EM: electron microscopy
ER: endoplasmic reticulum
ERES: ER exit site
E‐Syts: extended synaptotagmins
ERMES: ER–mitochondria encounter structure complex
IM: isolation membrane
LPS: lipopolysaccharide
LT: lipid transfer
LUV: large unilamellar vesicle
MT: membrane tethering
NR: N‐terminal region
PAS: preautophagosomal structure
PE: phosphatidylethanolamine
PI3K: phosphatidylinositol 3‐kinase
PI3P: phosphatidylinositol 3‐phosphate
PROPPIN: β‐propellers that bind polyphosphoinositides
SMP: synaptotagmin‐like mitochondrial lipid‐binding protein
Sp: *Schizosaccharomyces pombe*
SUV: small unilamellar vesicle
WIPI: WD repeat domain phosphoinositide‐interacting proteins

## Introduction

Autophagy is a highly conserved catabolic pathway in eukaryotes and occurs by a process of *de novo* organelle biogenesis.^1^, ^2^ In the budding yeast, *Saccharomyces cerevisiae*, this process starts with the nucleation of an isolation membrane (IM) in the vicinity of a vacuole. The cup‐shaped IM expands and encapsulates various cellular components, including biomolecules such as proteins and nucleic acids, and organelles such as mitochondria, endoplasmic reticulum (ER), and nucleus.^3^, ^4^, ^5^, ^6^ Finally, the IM becomes a double‐membrane organelle, termed an autophagosome, following the closure of its open mouth. The autophagosome fuses with the vacuole (known as a lysosome in mammals) and releases its inner membrane structure (the autophagic body) into the lumen of the vacuole, whereby the autophagic body together with its inner contents is degraded by vacuolar hydrolases for recycling the components. Autophagosome formation is an extremely important event in autophagy because the autophagy degradation targets are determined during this step. Autophagosome formation has attracted broad interest among researchers specialized in the field of autophagy as well as among cell biologists because this process is rare in the biogenesis of *de novo* double‐membrane organelles.

**Figure 1:**
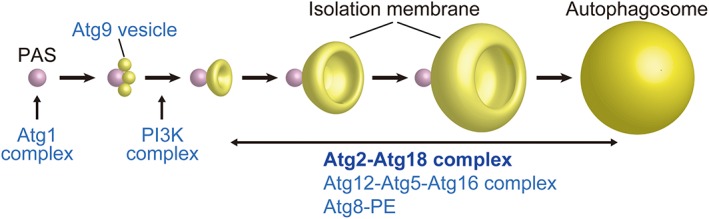
Proposed model of autophagosome formation in yeast. Upon autophagy induction, the Atg1 complex consisting of Atg1, Atg13, Atg17, Atg29, and Atg31 forms a higher‐order assemblage that functions as a scaffold for the preautophagosomal structure (PAS), the site of autophagosome formation.^10^, ^11^ Atg9 vesicles, which are 30–60 nm in size and contain a transmembrane protein Atg9, are generated from the Golgi apparatus and target to the PAS to become the IM precursor.^12^ The PI3K complex is then recruited to the PAS and produces PI3P in the IM precursor, which further recruits the Atg2–Atg18 complex and two ubiquitin‐like conjugation systems (Atg8–PE conjugate and Atg12–Atg5–Atg16 complex),^14^ leading to the expansion and closure of the IM into the autophagosome.

In budding yeast, dozens of autophagy‐related (Atg) proteins regulate the steps of autophagy through interaction with membranes.^7^ Of these proteins, 18 are essential for autophagosome formation during starvation‐induced autophagy.^1^ These essential proteins, termed core Atg proteins, are classified into the following six functional groups: the Atg1–protein kinase complex, Atg2–Atg18 complex, sole transmembrane protein Atg9, autophagy‐specific phosphatidylinositol 3‐kinase (PI3K) complex, Atg12–Atg5 conjugation system, and Atg8–phosphatidylethanolamine (PE) conjugation system.^8^ The current model of autophagosome formation mediated by these six groups is as follows (Fig. 1): upon autophagy induction, the Atg1 complex functions as a scaffold for constructing the autophagosome formation site, known as the preautophagosomal structure in yeast, and at the same time phosphorylates proteins, including Atg2 and Atg9.^9^, ^10^, ^11^ The Atg9‐containing vesicle, in collaboration with the Atg1 complex, generates an IM precursor, in which the PI3K complex produces phosphatidylinositol 3‐phosphate (PI3P), which then recruits the Atg2–Atg18 complex and two ubiquitin‐like conjugation systems for the expansion and closure of the IM into an autophagosome.^1^, ^12^, ^13^, ^14^ The conservation of the six functional groups among eukaryotes from yeasts to mammals suggests that the basic mechanism of autophagosome formation is evolutionarily conserved.

During the expansion of the IM, the Atg2–Atg18 complex localizes to the edge of the IM in a process dependent on PI3P and Atg9. The PROPPIN (β‐propellers that bind polyphosphoinositides) protein Atg18 recognizes PI3P for the recruitment of the Atg2–Atg18 complex to the IM,^15^ whereas the interaction of Atg9 with Atg2 restricts the localization of the Atg2–Atg18 complex to the edge of the IM.^16^ At the edge of the IM, the Atg2–Atg18 complex contacts the ER exit site (ERES) by an unknown mechanism, forming a contact site between the IM and ERES.^17^, ^18^ Although the physiological implications of the IM–ERES contact and the molecular functions of Atg2 had not been understood, recent advances in the structural and biochemical studies of Atg2 shed light on the critical function of Atg2 in autophagosome formation. This short review focuses on the recent advances in structural and biochemical studies of Atg2 and discusses the molecular mechanisms of the expansion of the IM, one of the biggest mysteries in the field of autophagy.

## A Brief History of Atg2 Analysis in the Prestructural Era

In 1993, the screening of *S. cerevisiae* autophagy‐defective mutants led to the identification of 13 *apg* genes required for autophagy, including *apg2*, later renamed to *atg2*.^19^ Eight years later, the *atg2* gene was cloned, and the essential role of the gene product, Atg2 protein, in autophagosome formation was established.^20^, ^21^ At that time, the affinity of Atg2 to the membranes was already implicit in its behavior as a peripheral membrane protein. However, the molecular functions of Atg2 were not elucidated for another 10 years. In 2012, studies on a mammalian Atg2 homolog, ATG2A, revealed that the C‐terminal portion of ATG2A is required for the localization of this protein to lipid droplets,^22^ and the lipid droplet localization was later confirmed by another group.^23^ These findings suggested that Atg2 has some affinity to lipids, but its significance in autophagy remains unclear. In 2013, Atg2 was shown to localize to the IM–ERES contact site,^17^, ^18^ an observation which indicated a critical role of Atg2 in autophagosomal membrane expansion. In 2017, the movement of the ER‐staining dye R18 from the ER to IM precursors was shown to be completely blocked by the deletion of Atg2,^24^ further suggesting a critical role of Atg2 in supplying lipids to the IM, although the molecular mechanisms involved remained unknown.

## Shape of the Atg2–Atg18 Complex

**Figure 2:**
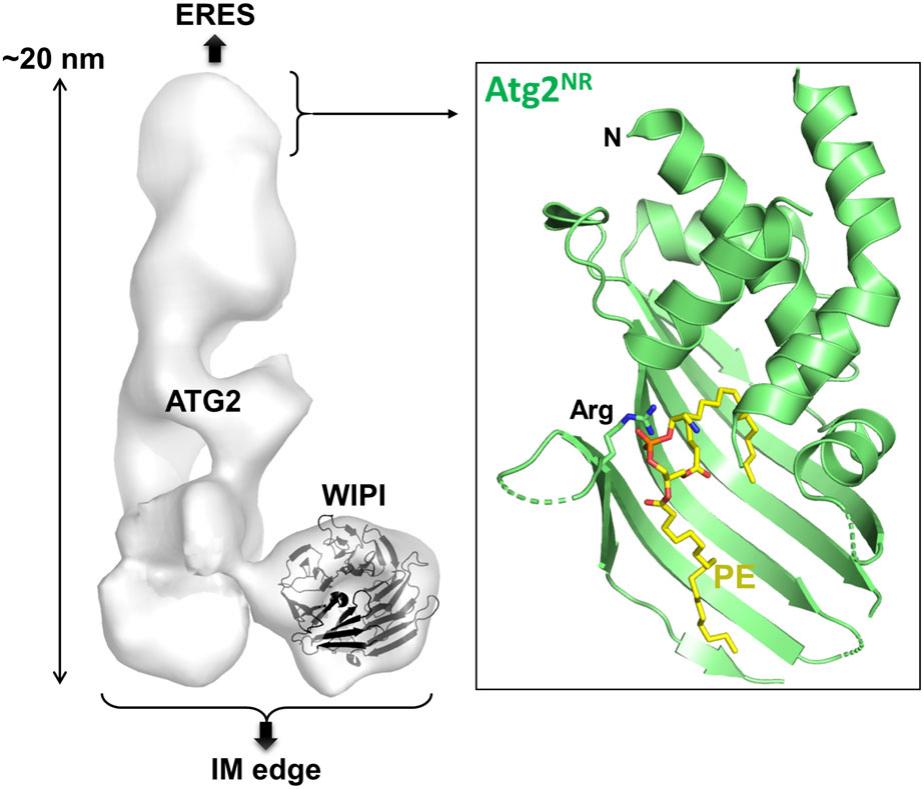
Structure of Atg2. Left, shape of the ATG2A–WIPI4 complex revealed by negative‐staining EM (EMDB ID 8899).^28^ Ribbon model represents the crystal structure of WIPI3 (PDB ID 6IYY)^35^ manually fitted into the EM image. Atg2‐family proteins have a rod‐like shape with ~20 nm in length and bind to ERES using one end and to IM using the opposite end, thereby tethering ERES to IM. Right, crystal structure of SpAtg2^NR^ (ribbon model) complexed with PE (stick model) (PDB ID 6A9J).^41^ PE is bound to the hydrophobic cavity of SpAtg2^NR^ using the acyl chains. The side‐chain of the arginine residue that interacts with the phosphoryl group of PE is shown with a stick model. Nitrogen and oxygen atoms are colored blue and red, respectively. N represents the N terminus.

Although structural studies on the core Atg proteins have significantly progressed in the last 15 years,^25^, ^26^, ^27^ the structure of Atg2 remained unsolved until 2017 due to its large size, 180 kDa in budding yeast, and the tendency of proteins to aggregate, making crystallization and NMR analysis difficult. However, recent advances in electron microscopy (EM) enabled the imaging of the mammalian Atg2–Atg18 complex at a low resolution.^28^, ^29^ Mammals have two Atg2 homologs (ATG2A and 2B) and four Atg18 homologs, known as WD repeat domain phosphoinositide‐interacting proteins (WIPI) 1–4.^22^, ^30^ Negative‐staining EM analysis of the ATG2A–WIPI4 complex (WIPI4 is also known as WDR45) and the ATG2B–WIPI4 complex revealed the overall shape of this protein complex (Fig. 2, left).^28^, ^29^ ATG2 has a rod‐like structure with a length of approximately 20 nm. The N terminus of ATG2 corresponds to one end of the rod, and the middle region of ATG2 forms the other end of the rod, binding to WIPI4 using an aromatic Y/HF motif.^29^ The C‐terminal half appears to go back to the N terminus, thereby producing an antiparallel topology, although the precise location of the C terminus of ATG2 in the rod structure is unclear due to its flexible conformation. Atg18 homologs belong to the PROPPIN family, which has a seven‐bladed β‐propeller fold with two phosphoinositide‐binding pockets that have been clearly characterized using high‐resolution crystal structures of Atg18 homologs.^31^, ^32^, ^33^, ^34^, ^35^ The β‐propeller ring of WIPI4 binds to one end of the ATG2 rod. Thus, the overall shape of the ATG2–WIPI4 complex looks like a golf club.^28^, ^29^ This architecture is roughly similar to a multiple‐subunit tethering complex, such as homotypic fusion and vacuole protein sorting,^36^ but the lack of high‐resolution structural information for Atg2 hampers further elucidation of the molecular functions of this complex family during autophagosome formation.

## Establishment of the Membrane‐Tethering Activity of Atg2

In parallel with the structural studies, the biochemical studies of Atg2 using purified proteins began to unveil the specific functions of Atg2. Purified ATG2B was found to bind to liposomes in a PI3P‐independent manner, in contrast to purified WIPI4 that requires a relatively large amount (∼15%) of PI3P for liposome binding.^29^ In these experiments, the membrane curvature of the liposomes did not affect the interaction. In addition, purified ATG2A was shown to bind to liposomes in a PI3P‐independent manner.^28^ In contrast to ATG2B, however, the size of liposomes markedly affected their affinity to ATG2A. ATG2A was strongly bound to small unilamellar vesicles (SUVs) but less strongly to large unilamellar vesicles (LUVs). Importantly, the size of SUVs was increased in the presence of ATG2A, but not when proteinase K was added, suggesting that ATG2A exhibits membrane‐tethering (MT) activity.^28^ Moreover, the ATG2A–WIPI4 complex could mediate the tethering of LUVs containing PI3P. EM analysis of the ATG2A–WIPI4 complex with SUVs revealed that one ATG2A binds to two liposomes using both the ends, thereby bridging them,^28^ further confirming that ATG2A alone exhibits MT activity. The MT activity was observed for *S. cerevisiae* Atg2 as well.^37^ Purified Atg2 showed binding to SUVs but not to LUVs in a PI3P‐independent manner. Moreover, purified Atg2 showed an MT activity for SUVs. All these activities are consistent with those observed for ATG2A. Truncation analysis identified two membrane‐binding regions in Atg2, one at the N terminus, involving residues 2–21, and the other at the C terminus, involving residues 1347–1592.^37^ Although each binding site alone was sufficient for binding to SUVs, both sites were required for tethering SUVs, indicating that Atg2 tethers SUVs using these two binding regions. Both binding regions were shown to be important for autophagy.^37^ Furthermore, the existence of two membrane‐binding regions in ATG2A was suggested by truncational analysis *in vivo*.^38^ The IM edge and ERES assume a high curvature^39^ and PI3P is enriched in the IM.^40^ Moreover, the N‐terminal portion of Atg2 was shown to be sufficient for targeting green fluorescent protein (GFP) to the ER.^37^ These observations suggest that the Atg2–Atg18 complex binds to both IM and ERES, thereby tethering them to each other.

## Atomic Resolution Structure of the N‐Terminal Region of Atg2

To date, although the full‐length Atg2 has not been crystallized, the N‐terminal region of *Schizosaccharomyces pombe* Atg2 (SpAtg2^NR^), which contains an evolutionarily conserved region termed Chorein_N, was recently crystallized and its crystal structure was determined.^41^ SpAtg2^NR^ comprises a helical region and a twisted β‐sheet, which fold into a globular structure with a large hydrophobic cavity. Mixing SpAtg2^NR^ and PE resulted in the formation of a SpAtg2^NR^–PE complex, whose structure was determined at 2.7 Å resolution (Fig. 2, right).^41^ A single PE molecule binds to SpAtg2^NR^ with its acyl chains buried within the hydrophobic cavity of SpAtg2^NR^, whereas the head group of the PE is exposed to the solvent. The phosphoryl group of the PE specifically interacts with the arginine residue of SpAtg2^NR^ although this electrostatic interaction is not required for autophagy. The phospholipid‐binding mode of SpAtg2^NR^ resembles that of synaptotagmin‐like mitochondrial lipid‐binding protein (SMP) domains, despite little topological similarity between them. The SMP domain is found in proteins, such as the ER–mitochondria encounter structure (ERMES) complex and extended synaptotagmins, both of which are localized at membrane contact sites and appear to transfer phospholipids between organelles.^42^ Moreover, SpAtg2^NR^ showed high structural similarity to the N‐terminal region of Vps13, which also contains Chorein_N.^43^ Vps13 is a multifunctional protein that has been suggested to mediate lipid transfer (LT) at various organelle contact sites.^44^ These observations strongly suggest that Atg2 is an LT protein that functions at the IM–ERES contact site.

## Discovery of the Phospholipid Transfer Activity of Atg2

LT assays using liposomes that contain fluorescently labeled phospholipids have demonstrated that Atg2 transfers phospholipids.^41^ The size of liposomes is important for the LT activity, and Atg2 shows strong LT activity only among SUVs. Considering that the tethering activity of Atg2 is restricted to SUVs, it appears that Atg2 transfers phospholipids between liposomes that are tethered to each other by the same Atg2 molecule. Most of the known LT proteins, such as those with SMP domains, have been experimentally shown to transfer lipids without tethering membranes. In terms of the close relationship between MT and LT activities, Atg2 is a novel type of LT protein. Atg18 itself showed no LT activity, but promoted the LT activity of Atg2, and in the presence of Atg18, PI3P further enhanced the LT activity of Atg2.^41^ Because the LT activity of Atg2 is proportional to its MT activity, Atg18 may promote the LT activity of Atg2 by enhancing its MT activity. Considering that Atg2 localizes to the IM–ERES contact site, it is proposed that Atg2 mediates tethering of the IM to ERES and simultaneously transfers phospholipids between them.

*In vitro* LT activity was also confirmed for human ATG2A^45^, ^46^ and ATG2B (Osawa and Noda, unpublished observation). ATG2A bridges SUVs and transfers phospholipids between them, which is accelerated by the addition of PI3P together with WIPI proteins.^45^ Moreover, ATG2A was shown to localize to autophagosome–ER contact sites in mammalian cells.^46^ These observations are consistent with those observed for yeast Atg2, suggesting that the LT and MT activities of Atg2 and their role in autophagy are evolutionarily conserved.

## Role of Atg2^NR^ in Phospholipid Transfer

Atg2^NR^ binds to a variety of phospholipids, including PE, phosphatidylcholine, and phosphatidylserine with low specificity.^41^ Conversely, the LT activity of Atg2^NR^ is much lower than that of the full‐length Atg2, implying that a region other than Atg2^NR^ is required for efficient phospholipid transfer. Together with the observation that Atg2^NR^ is located at the one end of the rod‐like architecture and Atg2 tethers two membranes using both ends, it is proposed that Atg2^NR^ functions as an extractor of phospholipids from one membrane. The substitution of the conserved basic residues at the surface of Atg2^NR^ with acidic residues caused significant reduction in the MT and LT activities of Atg2.^41^ Although the basic residues of Atg2^NR^ are not conserved in Vps13, a chimeric Atg2 involving the Chorein_N of Vps13, instead of the intrinsic Chorein_N, exhibited the same autophagic activity as wild‐type Atg2.^41^ The Chorein_N regions of Atg2 and Vps13 possess high structural similarity to each other and conserve a hydrophobic cavity despite the low sequence similarity.^41^, ^43^ Chorein_N may function to extract phospholipids from membranes as a prerequisite to LT. In humans, the dysfunction of VPS13A causes a neurodegenerative disorder, Chorea‐acanthocytosis.^47^ Some of the missense mutations in *VPS13A*, which are found in Chorea‐acanthocytosis patients, are located in the Chorein_N region,^48^ suggesting that a defect of phospholipid transfer at organelle contact sites may be one of the causes of some neurodegenerative disorders.

## Proposed Mechanism of Phospholipid Transfer Mediated by Atg2

**Figure 3:**
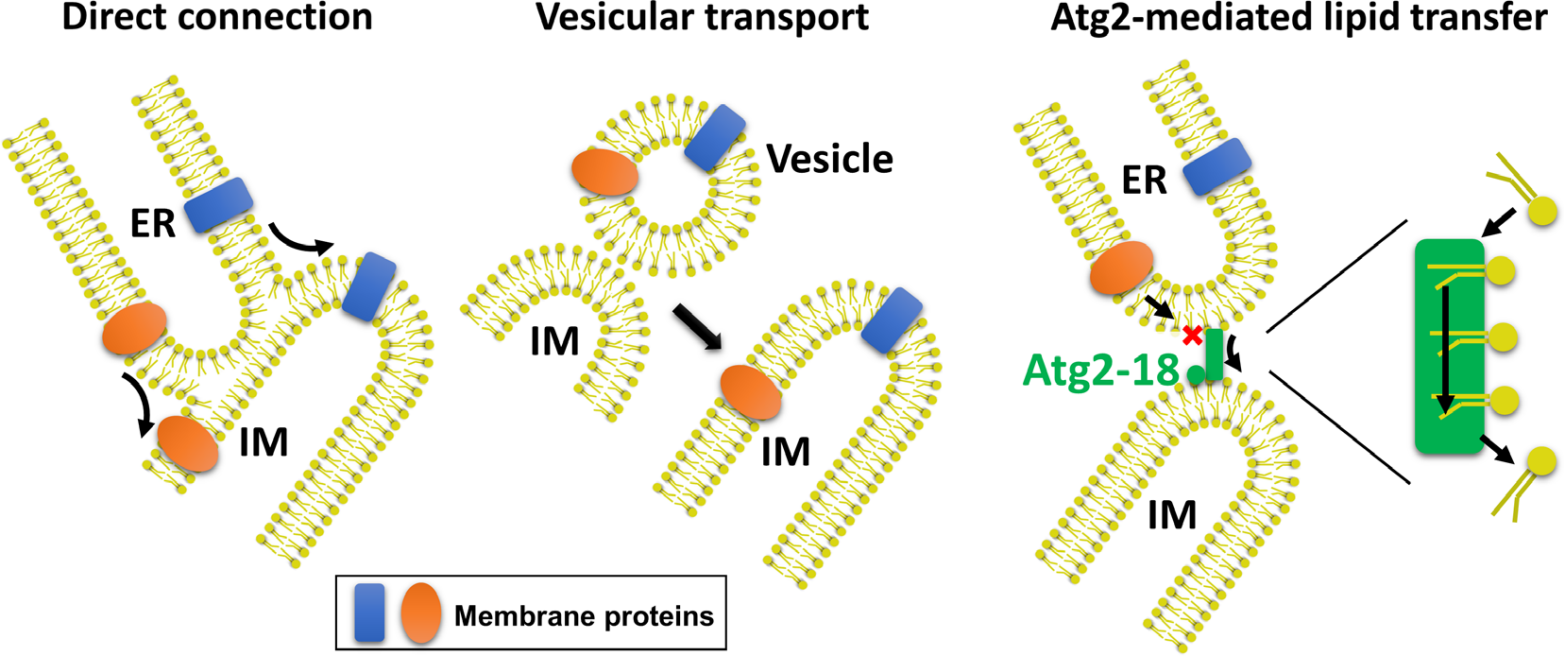
Distinct models of supplying phospholipids for autophagosome formation. Left, direct connection model. Phospholipids in the ER move to the IM through direct connection. Middle, vesicle‐mediated model. Fusion of vesicles derived from ER or other membrane source organelles with the IM provides phospholipids. Right, Atg2‐mediated model. Phospholipids in the ER move to the IM through Atg2. Among these models, the Atg2‐mediated model seems to be suitable for transferring phospholipids while excluding the influx of membrane proteins, which are abundant in ER and other membrane source organelles, to the IM.

How does Atg2 transfer phospholipids from ERES to the IM? Because the MT activity is indispensable for the LT activity of Atg2, a well‐known LT system, in which LT proteins shuttle between organelles,^49^ does not appear to apply to Atg2. As described above, Atg2‐mediated LT requires the region downstream of Atg2^NR^, which has been predicted to contain five repeated structures.^41^ These repeated structures are formed mainly by β‐strands rich in hydrophobic residues, suggesting that they might have a fold similar to the β‐jellyroll that harbors a hydrophobic groove. Among the five repeats, the most C‐terminal repeat contains a conserved ATG2_CAD region, which appears, from EM analyses, to form the IM‐binding surface together with Atg18.^28^ An attractive model is that Atg2^NR^ and the five repeated structures constitute a successive hydrophobic groove that functions as a pathway for phospholipids; phospholipids move from ERES to the IM by sliding the hydrophobic acyl chains along the hydrophobic groove while exposing the hydrophilic head groups to the cytosol (Fig. 3, right). Indeed, recent Cryo‐EM analysis of ATG2A at 15 Å resolution proposed the existence of hydrophobic grooves or cavities throughout the rod structure although their consecutiveness remained elusive.^46^ A long hydrophobic groove has been observed in other LT protein complexes. The Lpt complex comprises seven components, named LptA–G, and it transfers lipopolysaccharides (LPSs) across the periplasm in bacteria.^50^ In the Lpt complex, LptA, LptC, and LptD are equipped with β‐jellyroll domains, which stack upon each other to form a hydrophobic pathway, that enable LPS molecules to cross the periplasm. The ERMES complex comprises four subunits (Mdm12, Mdm35, Mdm10, and Mmm1) and mediates phospholipid transfer between mitochondria and the ER.^51^ Mdm12 and Mmm1, both of which possess SMP domains, form a tubular tetramer in a 2:2 manner, which produce a successive hydrophobic cleft through the four molecules although the significance of the long cleft remains to be established.^52^ These examples suggest that a long hydrophobic groove within a protein or protein complex could function as a pathway for LT. It is necessary to experimentally validate whether Atg2 has a similar long hydrophobic groove and the groove, if it exists, mediates phospholipid transfer between membranes that are bridged by Atg2.

To expand the IM, Atg2 must mediate a unidirectional transfer of phospholipids from ERES to IM. Atg18 and Atg9 might determine the flow direction of the phospholipids by fixing the orientation of Atg2: Atg18 binds to one end of Atg2, which is opposite to Atg2^NR^, and recognizes PI3P existing in the IM.^15^, ^28^, ^29^ Atg9 localizes to the edge of the IM and interacts with Atg2 at the edge.^16^ These interactions localize the Atg2–Atg18–Atg9 complex at the edge of the IM and, at the same time, orient Atg2^NR^ toward ERES, possibly allowing Atg2^NR^ to interact with ERES but not with IM and extract phospholipids only from ERES.

## Proposed Mechanism of *De Novo* Autophagosome Biogenesis

Autophagosomes, 0.3–0.9 μm in size, are formed within 10 min in yeast.^53^, ^54^, ^55^ Compared with other organelle membranes, autophagosomal membranes contain few membrane proteins.^56^ Because the role of autophagosomes is restricted to deliver substrates to lysosomes/vacuoles for degradation, a minimum number of membrane proteins would be sufficient for the functioning of autophagosomes. Several mechanisms have been proposed for membrane supply during autophagosome formation, including direct connections between the IM and ER, and vesicular transport (Fig. 3).^57^, ^58^, ^59^, ^60^ One of the biggest shortcomings of these models is that it would be difficult to supply lipids while excluding membrane proteins that are abundant in membrane source organelles, including the ER. An Atg2‐mediated lipid supply appears more likely because Atg2 could transfer lipids while blocking the influx of membrane proteins to the IM. To construct the autophagosomal membranes, significant amounts of phospholipid molecules (more than a million) are required as building blocks. Currently, it is unclear what percentage of phospholipids in an autophagosome is provided by the LT activity of Atg2. If most phospholipids are provided by the LT activity of Atg2, Atg2 must mediate a fairly rapid transfer of phospholipids from the ER and other membrane source organelles to the IM. Because Atg2 itself does not use energy for LT, mechanisms including other unknown proteins would be required for ensuring the unidirectional, highly efficient phospholipid transfer that is needed for *de novo* autophagosome biogenesis.

## Conclusions

Recent advances in structural biology and biochemical studies have shed light on the molecular functions of Atg2, one of the most poorly understood proteins in the field of autophagy. Thus, the finding that Atg2 is a novel type of phospholipid transfer protein, bridging two membranes and at the same time transferring phospholipids between them, is unexpected and provides a novel model of autophagosome formation: LT proteins supply phospholipids from membrane source organelles as building blocks for autophagosome formation. Although technically difficult, *in vivo* visualization of phospholipid movement from the ER to IM via Atg2 and atomic resolution structural determination of the full‐length Atg2 will provide further mechanistic insights into Atg2 function solving one of the biggest questions in autophagy: the mechanism of *de novo* autophagosome biogenesis.
